# TGF-β1-Smad signaling pathways are not required for epidermal LC homeostasis

**DOI:** 10.18632/oncotarget.8167

**Published:** 2016-03-17

**Authors:** Guihua Li, Xing-hua Gao, Qing-Sheng Mi

**Affiliations:** Department of Dermatology, No. 1 Hospital of China Medical University, Shenyang, P.R. China

**Keywords:** langerhans cells, TGF-β, Smad2, Smad3, Smad4, Gerotarget

Langerhans cells (LCs) are skin-homing dendritic cells (DCs) that have long been considered to be prototypic sentinel DCs due to their prominent position at the environmental barrier, and are essential for the induction of skin immunity and tolerance [1]. Transforming growth factor-β1 (TGF-β1) is a key factor in epidermal LC development and maintenance. The presence of TGF-β1 is a prerequisite for in vitro LC differentiation from various sources. For examples, human CD34^+^ hematopoietic progenitor cells expanded and developed into LCs in a stringent TGF-β1-dependent manner under serum-free conditions. Both TGF-β1 and TGF-β receptor II (TGF- βRII) null mice exhibited a profound LC loss in skin [2]. Furthermore, LCs are absent in mice that lack Id2 and Runx3, the transcription factors controlled by the TGF-β pathways. In the DC-specific TGF-βRI knockout (KO) mice, LCs disappeared within the first week after birth and displayed increased expressions of costimulatory and pro-motility molecules, suggesting that TGF-β1 is also required to maintain the immature status of epidermal LCs [3]. Latest studies also suggested that TGF-β1 inhibits steady-state and inflammation-induced LCs maturation and migration [4].

Despite recent great advances in understanding of LC homeostasis and function mediated by TGF-β1, the underlying mechanisms and TGF-β signaling pathways remain elusive. Canonically, binding of TGF-β to TGF- βR activates two structurally similar proteins, receptorassociated Smads, Smad2 and Smad3 in the target cells. Activated Smad2 or Smad3 hetero-dimerizes with Smad4 and then translocate into the nucleus acting as transcriptional regulators of TGF-β1 target genes. In addition, Smad-independent pathways also exist to mediate the TGF-β signaling. By genetic approaches, we investigated if Smad-dependent pathways are required for epidermal LC maintenance after birth. Using Smad3 KO mice, our previous study suggested that TGF-β/Smad3 signaling pathway is not required for LC homeostasis [5]. This raises a possibility that Smad2 may be involved in TGF-β1-mediated LC development. Given the embryonic lethality in the conventional Smad2-deficient mice, we crossed human Langerin-*Cre* transgenic mice [4] with Smad2*^fl/fl^* mice to generate LC-specific Smad2 conditional KO mice. Interestingly, like Smad3 deficiency, Smad2 deficiency did not significantly affect epidermal LC homeostasis after birth and is not required for maintaining LC immaturation at steady state and not for regulating LC maturation upon *in vitro* culture. Smad4 is a key player in the downstream of TGF-β1-mediated Smad pathway. To further dissect TGF-β/Smad pathway in LC development, we next generated the mice with LC-selective ablation of Smad4. Surprisingly, similar to what we found in Smad2KO mice, Smad4 deficiency did not affect the LC ratio and immature station in the epidermis. Thus, Smad4 deficiency doesn't significantly interrupt LCs homeostasis and maturation.

**Figure 1:**
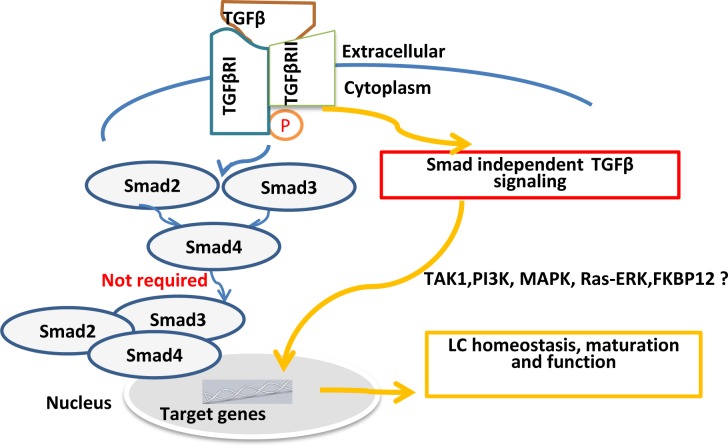
TGF-β1-Smad signaling pathways are not required for epidermal LC homeostasis

In conclusion, in combination with our previous findings from Smad3KO mice [5], our recent findings highly suggest that conventional TGF-β/Smad2/3/4 signaling pathways may not be required for epidermal LC homeostasis (Figure 1). It is increasingly apparent that TGF-β not only activates Smads but also actives other non-Smad signaling pathways, including rapid activation of TGF-β-activated kinase 1 (TAK1), Ras-Erk, PI3K-Akt pathway, FKBP12, TRIP-1, late endosomal adaptor molecule p14 (LAMTOR2) and eIF2a [7, 8]. The underlying mechanisms by which TGF-β/Smadindependent pathways regulate LCs maintenance and function remain to be further determined.
